# Optical and thermoelectric properties of new Janus ZnMN_2_ (M = Ge, Sn, Si and N = S, Se, Te) monolayers: a first-principles study

**DOI:** 10.1039/d3na00905j

**Published:** 2023-12-05

**Authors:** Basit Ali, Muhammad Idrees, Tahani A. Alrebdi, Bin Amin, Qaisar Alam

**Affiliations:** a Department of Physics, Abbottabad University of Science and Technology Abbottabad Pakistan qaisaralam96@gmail.com +92-346-833-4932; b Department of Physics, College of Science, Princess Nourah Bint Abdulrahman University P.O. Box 84428 Riyadh 11671 Saudi Arabia

## Abstract

Thermoelectric materials have received great interest because they directly tap into the vast reserves of currently underused thermal energy, in an environmentally friendly manner. In this work, we investigated the electronic, optical and thermoelectric properties of novel ZnMN_2_ (M = Ge, Sn, Si and N = S, Se, Te) monolayers by performing density functional theory calculations. The dynamic and thermal stabilities of ZnMN_2_ (M = Ge, Sn, Si and N = S, Se, Te) monolayers were confirmed by their phonon band structures and *ab initio* molecular dynamics (AIMD) simulations, which showed that all the studied monolayers are stable. Calculated electronic band structures showed that ZnSiTe_2_, ZnGeSe_2_, and ZnSnTe_2_ have a direct band gap, while the remaining monolayers have an indirect band gap. Optical properties in terms of the imaginary part of the dielectric function have also been investigated, which showed that all the first excitonic peaks lie in the visible region. Transport coefficients, such as the Seebeck coefficient (*S*), electrical conductivity (*σ*) and power factor (PF) were calculated using the Boltzmann theory and plotted against chemical potential. The results demonstrated that the peak values of the p-type region for the PF are greater than those of the n-type region. Notably, ZnSiTe_2_ exhibits a large PF due to its smaller Seebeck coefficient and higher electrical conductivity compared to ZnSnS_2_, indicating that it is a promising candidate for thermoelectric applications. Our findings reveal that ZnMN_2_ (M = Ge, Sn, Si and N = S, Se, Te) monolayers open up new possibilities for optoelectronics and thermoelectric device applications.

## Abstract 2

In this work, we investigated the electronic, optical and thermoelectric properties of novel ZnMN_2_ (M = Ge, Sn, Si and N = S, Se, Te) monolayers by performing density functional theory calculations..

## Graphical abstract

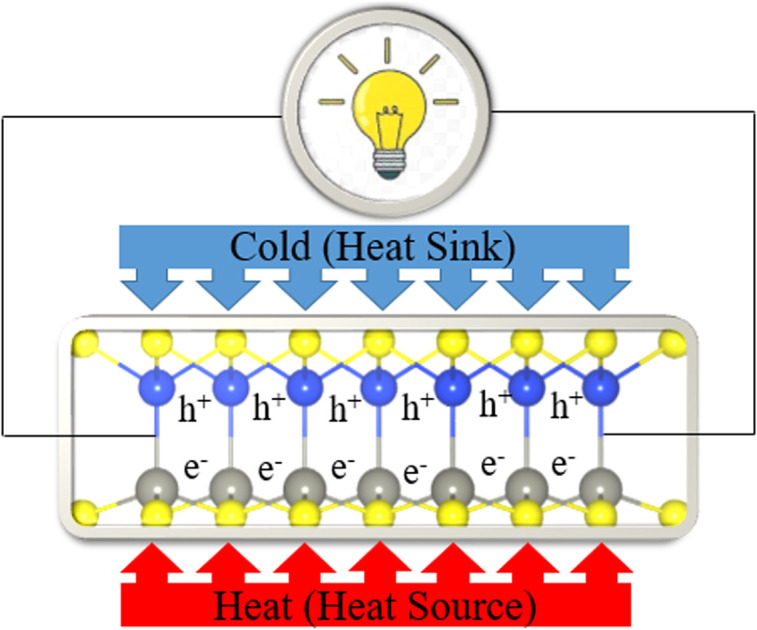

## I. Introduction

A shortage of energy is currently predicted to be a great challenge in the near future for our society.^1^ It is expected that the world’s energy demands will rise by up to 60% by 2030.^1^ Currently, about 80% of the world’s energy is generated by heat engines using fossil fuels as an energy source, which has a big drawback of carbon dioxide emission.^1^ Due to this increasing demand for clean energy, researchers are trying to develop renewable energy sources to resolve energy issues and also to avoid environmental pollution.^2,3^ Recently, solar cells, wind-driven generators, thermoelectric devices and fuel cells have attracted tremendous attention. Among these resources, thermoelectric devices have gained much attention due to their many applications, including cooling devices, sensors and power generators.^4–7^ Thermoelectric materials have the ability to directly convert thermal energy, such as solar and waste energy, to electrical energy, which makes them suitable for development into sustainable energy devices.^8–10^ In comparison with other energy sources, thermoelectric devices have advantages of stability, silent operation and long service life.^11–13^ The efficiency of thermoelectric materials depends on the Seebeck coefficient (*S*), electrical conductivity (*σ*), total conductivity (*K*) and absolute temperature (*T*). For efficient thermoelectric materials, the electrical conductivity (*σ*) and Seebeck coefficient (*S*) is high and the thermal conductivity is low.^14,15^

Since the discovery of single-layer graphene in 2004,^16^ research interest in the field of 2D materials has grown explosively in just a few years. Recently, thanks to developments in synthesis techniques and theoretical simulation, a variety of 2D materials have been theoretically predicted and successfully fabricated, such as hexagonal boron nitride (h-BN),^17^ transition-metal dichalcogenides (TMDCs),^18^ group-III metal chalcogenides,^20^ group-IV and -VI metal monochalcogenides, such as SiO, SiS, GeS and SnS,^21^ and carbide and nitride semiconductors.^22^ Lately, a novel class of 2D materials named Janus transition-metal dichalcogenides (JTMDCs) has attracted attention from researchers due to the distinct properties that the materials obtain from their parent transition-metal dichalcogenide monolayers. Lu and co-workers^23^ confirmed the successful fabrication of JTMDCs by using chemical vapor deposition (CVD). Recently, a group of novel 2D Janus group-III monochalcogenides with the general formula ZnXY_2_ (X = Si, Ge, Sn and Y = S, Se, Te) and constructed according to their isoelectronic mutation has been investigated, and refers to group-III monochalcogenides,^24^ which have been attracting much interest due to their higher piezoelectricity, direct band-gaps and large effective mass difference, making them good candidates for applications in optoelectronics, photocatalysis, flexible nanodevices and electromechanical systems.^24^

Due to their appealing mechanical and electronic properties, 2D materials with layered structures have attracted tremendous attention as efficient thermoelectric materials. In previous decades, thermoelectric properties of 2D materials have been computationally investigated and the materials have also been experimentally fabricated.^25–27^ In addition, Guo *et al.*^28^ reported that the n-type Janus ZrSSe monolayer has excellent thermoelectric performance, which was better than that of a ZrS_2_ monolayer. According to Bera *et al.*, the HfSSe monolayer was dynamically stable, and gradual increases and decreases in the bandgap were observed with changes in the applied biaxial strain. They also suggested that, due to the SOC effect, the HfSSe monolayer has a higher power factor than the HfSe_2_ monolayer but lower than that of the HfS_2_ monolayer.^29^ These 2D materials exhibit fascinating properties, such as a large potential, when they are used in the fabrication of high-performance thermoelectric devices.

Although interesting research results for both experimental and theoretical prediction of thermoelectric properties have been calculated, a comprehensive study of ZnMN_2_ (M = Si, Ge, Sn and N = S, Se, Te) monolayers is still missing. So, here in this paper, we investigated the structural, optoelectronic and thermoelectric properties of ZnMN_2_ (M = Si, Ge, Sn and N = S, Se, Te) monolayers using density functional theory, which is based on first-principles calculations.

## II. Computational details

These calculations are based on density functional theory (DFT) techniques and approaches using PWSCF code.^30^ In the first Brillouin zone (BZ), a *Γ*-point-centered 12 × 12 × 1 Monkhorst–Pack *k*-point grid is used with a kinetic energy 450 eV. To avoid the interactions between adjacent layers of atoms, a vacuum layer with thickness of 25 Å is considered. Forces and energies are converged to 10^−3^ eV Å and 10^−6^ eV, respectively. The exchange-correlation functional is a key component that accounts for the electron–electron interactions in a system, so we used the GGA approximation.^31,32,37^ Boltzmann semi-classical theory^33^ is used to calculate the electrical transport properties, like the Seebeck coefficient (*S*), electrical conductivities (*σ*), thermal conductivities (*κ*) and power factor (PF), using the BoltzTraP software package. This is primarily designed for studying the thermoelectric and electronic transport properties of materials, such as electrical conductivity, the Seebeck coefficient, and the electronic density of states. BoltzTraP uses the Boltzmann transport theory and the constant relaxation time approximation.^33^ The following equations can be used to express all of these parameters:1
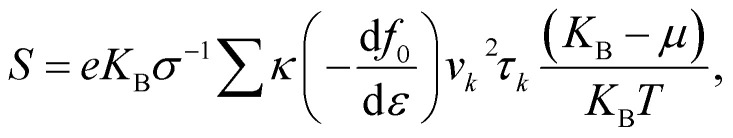
2
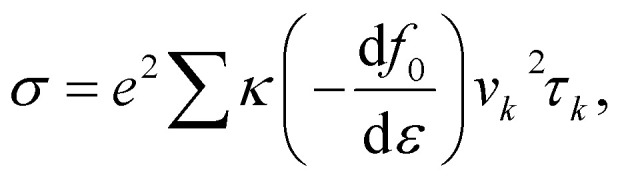


and the power factor (PF) is

3 PF = *S*^2^*σ*.

In the equations above, *e* is the charge on the elementary charge carrier, *ε* is the energy, *K*_B_ is the Boltzmann constant, *τ*_*k*_ is the relaxation time, *f*_0_ is the Fermi distribution function, *μ* denotes the chemical potential, and *v*_*k*_ is the group velocity.

## III. Results and discussion

**Fig. 1:**
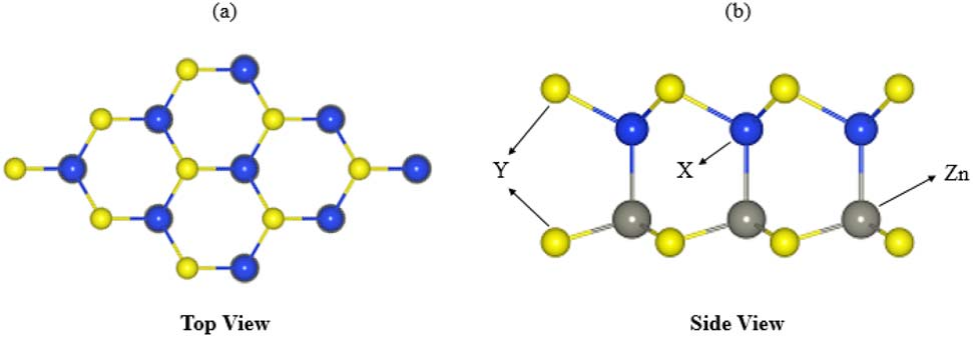
Top (a) and side (b) views of ZnMN_2_ (M = Si, Ge and Sn and N = S, Se and Te) monolayers.

Optimized lattice constants of ZnSiS_2_, ZnSiSe_2_, ZnSiTe_2_, ZnGeS_2_, ZnGeSe_2_, ZnGeTi_2_, ZnSnS_2_, ZnSnSe_2_ and ZnSnTi_2_ monolayers are listed in Table 1 and are in agreement with previously reported work.^19^ The geometrical structure of the ZnMN_2_ monolayer consists of four sublayers with two middle layers of Zn and M (M = Si, Ge, Sn), and two outer layers of N (N = S, Se, Te). The monolayer has a hexagonal structure and is held together by covalent bonds, as presented in Fig. 1, where the gray spheres represent the Zn atoms, the yellow spheres represent Si, Ge, or Sn, and the blue spheres represent different chalcogen atoms.^34,35^ One can easily observe that the bond lengths for the bonds containing S atoms are shorter than those containing Se and Te atoms due the difference in electron affinities of S, Se, and Te atoms. The calculated lattice constants, bond lengths and band gaps of the various ZnMN_2_ monolayers are given in Table 1.

**Table 1:** Calculated lattice constant (*A*), bond length (*d*) and band gap (*E*_g_) values of ZnMN_2_ (M = Si, Ge and Sn and N = S, Se and Te) monolayers

ZnMN_2_	*A* (Å)	*d*_*z*–*x*_ (Å)	*d*_Zn–*y*_ (Å)	*d*_*x*–*y*_ (Å)	*E*_g_ PBE (eV)	*E*_g_ HSE (eV)
ZnSiS_2_	3.710	2.465	2.243	2.414	0.276	0.73
ZnSiSe_2_	3.890	2.466	2.380	2.552	0.294	0.60
ZnSiTe_2_	4.190	2.410	2.578	2.745	0.195	0.35
ZnGeS_2_	3.790	2.661	2.235	2.494	1.950	1.90
ZnGeSe_2_	3.960	2.633	2.365	2.635	0.950	1.39
ZnGeTe_2_	4.250	2.547	2.561	2.820	0.750	1.03
ZnSnS_2_	3.870	2.979	2.253	2.633	1.590	2.43
ZnSnSe_2_	4.050	2.838	2.385	2.801	1.320	2.04
ZnSnTe_2_	4.330	2.684	2.531	2.899	0.476	1.63

**Fig. 2:**
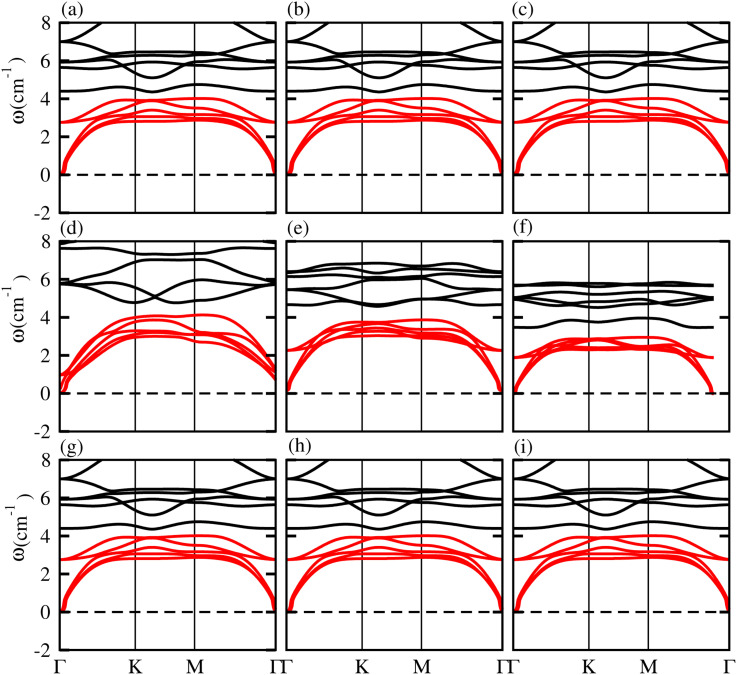
Phonon band spectra of (a) ZnSiS_2_, (b) ZnSiSe_2_, (c) ZnSiTe_2_, (d) ZnGeS_2_, (e) ZnGeSe_2_, (f) ZnGeTe_2_, (g) ZnSnS_2_, (h) ZnSnSe_2_, (i) ZnSnTe_2_, monolayers.

**Fig. 3:**
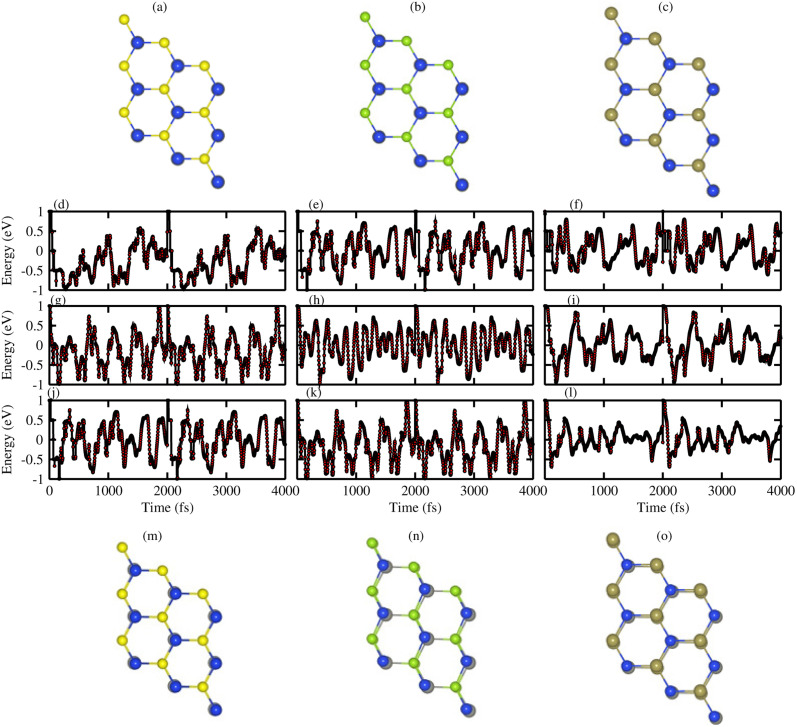
AMID calculation of ZnSiN_2_ (N = S, Se and Te) monolayers, where (a–c) before heating; (m–o) after heating, while ((d)–(l)) is the fluctuation energy of all ZnMN_2_ (M = Si, Ge and Sn and N = S, Se and Te) monolayers.

To investigate the dynamic stability of ZnMN_2_ (M = Ge, Sn, Si and N = S, Se, Te) monolayers, phonon band spectra were calculated using the phonopy code, as shown in Fig. 2. There are four atoms in each primitive unit cell, therefore, each phonon band dispersion is composed of three acoustic zero-frequency modes and 12 optical branches. It is clear from Fig. 2 that all phonon modes have positive eigenfrequencies at the *Γ*-point of the Brillouin zone, indicating that ZnMN_2_ (M = Ge, Sn, Si and N = S, Se, Te) monolayers are dynamically stable, which confirms that ZnMN_2_ (M = Ge, Sn, Si and N = S, Se, Te) monolayers can be experimentally synthesized in the laboratory. Furthermore, we investigated the thermal stability of these monolayers using *ab initio* molecular dynamics calculations. The results are presented in Fig. 3, confirm that there is no bond breaking at room temperature (300 K) to the system, hence these systems are stable at room temperature.^36,39^

**Fig. 4:**
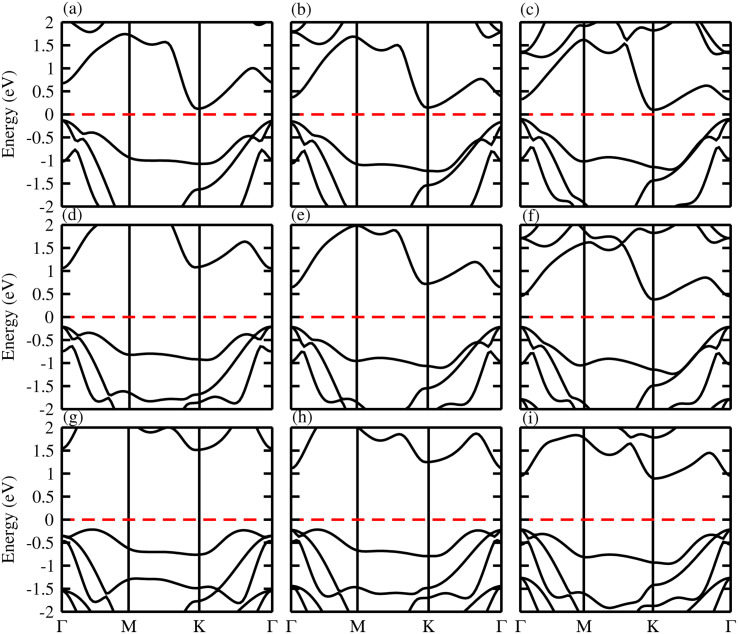
Calculated band structure of (a) ZnSiS_2_, (b) ZnSiSe_2_, (c) ZnSiTe_2_, (d) ZnGeS_2_, (e) ZnGeSe_2_, (f) ZnGeTe_2_, (g) ZnSnS_2_, (h) ZnSnSe_2_ and (i) ZnSnTe_2_ monolayers.

The band structure and band gap values are very sensitive to the choice of exchange correlation function, therefore, we considered both PBE and HSE06 functionals for our calculations. The calculated electronic band structures of ZnMN_2_ (M = Si, Ge, Sn; N = S, Se, Te) using the PBE^37,38^ functionals are shown in Fig. 4. One can see from the calculated electronic band structures that all the monolayers are semiconducting with a narrow band gap, and are therefore suitable for thermoelectric applications. One can also observe that ZnGeS_2_, ZnGeSe_2_ and ZnSnTe_2_ have a direct band gap with the CBM and VBM lying at the *Γ*-point, while the remaining monolayers have an indirect band gap with the VBM and CBM at the *Γ*- and *M*–*Γ*-points, respectively, of the first BZ. It is also observed that due to the chalcogen atom bonded to Zn and Si, the band gap values drop from S to Te. The calculated values are listed in Table 2 and are in good agreement with previous available data.^19^

**Table 2:** Calculated values of the Seebeck coefficient (*S*), electrical conductivity (*σ*) and power factor (PF) at 300 K and 800 K for the p-type doped region of ZnSiS_2_, ZnSiSe_2_, ZnSiTe_2_, ZnGeS_2_, ZnGeSe_2_, ZnGeTe_2_, ZnSnS_2_, ZnSnSe_2_ and ZnSnTe_2_ monolayers.

Monolayers	300 K			800 K		
	*S* (μV/K)	*σ* (1/Ω ms)	PF (Wm K^2^ s)	*S* (μV/K)	*σ* (1/Ω ms)	PF (Wm K^2^ s)
ZnSiS_2_	450	0.89 × 10^20^	0.59 × 10^11^	230	0.8 × 10^20^	1.2 × 10^11^
ZnSiSe_2_	500	0.95 × 10^20^	0.5 × 10^11^	250	0.9 × 10^20^	1.4 × 10^11^
ZnSiTe_2_	200	0.9 × 10^20^	0.55 × 10^11^	120	0.86 × 10^20^	1.5 × 10^11^
ZnGeS_2_	500	0.90 × 10^20^	1.00 × 10^11^	800	0.60 × 10^20^	2.00 × 10^11^
ZnGeSe_2_	1800	1.00 × 10^20^	0.70 × 10^11^	650	0.70 × 10^20^	2.50 × 10^11^
ZnGeTe_2_	1200	0.80 × 10^20^	0.50 × 10^11^	400	0.70 × 10^20^	2.20 × 10^11^
ZnSnS_2_	1000	0.90 × 10^20^	0.80 × 10^11^	350	0.80 × 10^20^	1.40 × 10^11^
ZnSnSe_2_	2000	0.70 × 10^20^	1.30 × 10^11^	800	0.60 × 10^20^	2.00 × 10^11^
ZnSnTe_2_	1400	0.40 × 10^20^	0.30 × 10^11^	400	0.30 × 10^20^	0.70 × 10^11^

**Fig. 5:**
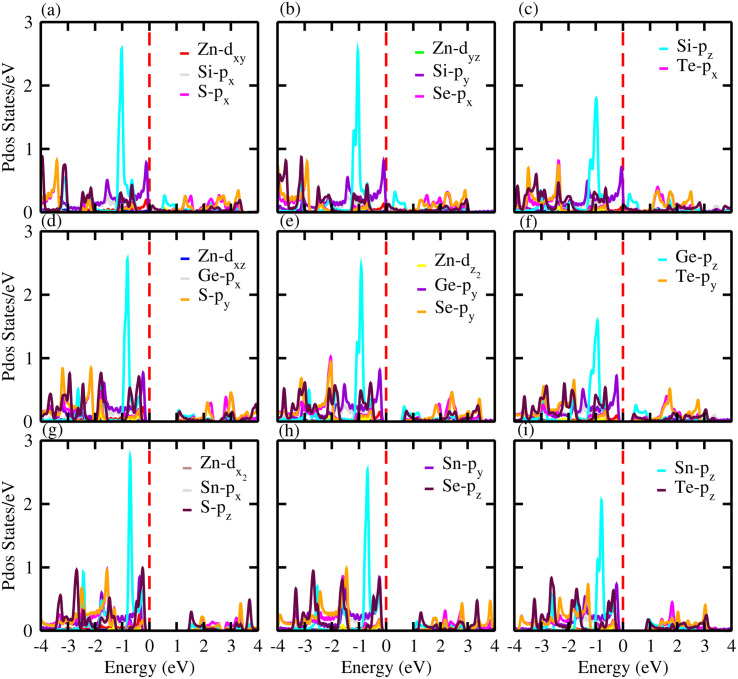
Partial density of states (PDOS) for (a) ZnSiS_2_, (b) ZnSiSe_2_, (c) ZnSiTe_2_, (d) ZnGeS_2_, (e) ZnGeSe_2_, (f) ZnGeTe_2_, (g) ZnSnS_2_, (h) ZnSnSe_2_ and (i) ZnSnTe_2_ monolayers in which the contributions of different atoms are labelled in the figure.

For further verification of the various atomic states of the monolayers, we computed the partial density of states (PDOS) of ZnXY_2_ monolayers, which are depicted in Fig. 5. The contributions of different states of the atoms are shown by different colors. From the PDOS of the ZnSiS_2_, ZnSiSe_2_ and ZnSiTe_2_ monolayers, it can be clearly seen that in the VBM the major contribution is due to the Te-p_*z*_ atom, whereas the CBM is generated by the S, Se and Te-p_*z*_ atoms. In the second row which shows results for the ZnGeS_2_, ZnGeSe_2_ and ZnGeTe_2_ monolayers, the VBM and CBM are due to the Ge and chalcogen p_*z*_ atoms, respectively. While in the third row, which shows results for ZnSnS_2_, ZnSnSe_2_ and ZnSnTe_2_ monolayers, the VBM and CBM are from the Sn-p_*z*_ and chalcogen p_*z*_ states. In all these monolayers, some little contributions are also present due to Zn atoms. High mobility may be favored by this type of partially ionic nature with cross-gap hybridization.^40^

**Fig. 6:**
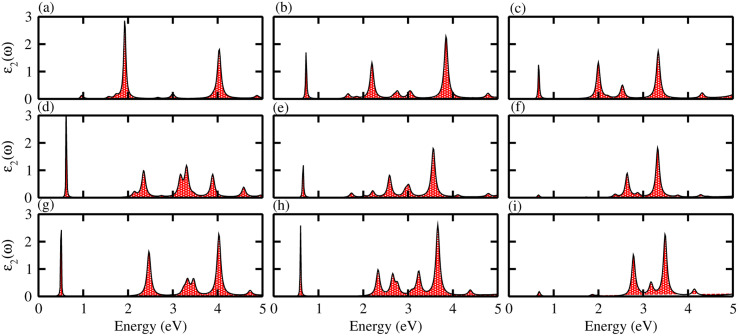
Optical spectra of (a) ZnSiS_2_, (b) ZnSiSe_2_, (c) ZnSiTe_2_, (d) ZnGeS_2_, (e) ZnGeSe_2_, (f) ZnGeTe_2_, (g) ZnSnS_2_, (h) ZnSnSe_2_ and (i) ZnSnTe_2_ monolayers.

The absorption spectra of systems are measured to investigate the optical properties of materials. They allow the dielectric function *ε*_2_(*ω*) ^46^ to be determined, therefore absorption spectra are a fundamental tool for understanding the interaction of electromagnetic radiation with matter. The imaginary part of the dielectric function *ε*_2_(*ω*) was calculated for the different monolayers and the results are shown in Fig. 6. Exciton peaks can clearly be seen for ZnSiS_2_ (0.96 eV), ZnSiSe_2_ (0.72 eV), ZnSiTe_2_ (0.662 eV), ZnGeS_2_ (0.61 eV), ZnGeSe_2_ (0.661 eV), ZnGeTe_2_ (0.64 eV), ZnSnS_2_ (0.52 eV), ZnSnSe_2_ (0.63 eV) and ZnSnTe_2_ (0.67 eV). These values indicate strong modifications in the positions of excitons from the S atom to the Te atom, suggesting that this is promising for controlling the exciton–phonon interactions or coupling at the nanoscale, and for applications in thermal imaging sensors in the future.^47^

**Fig. 7:**
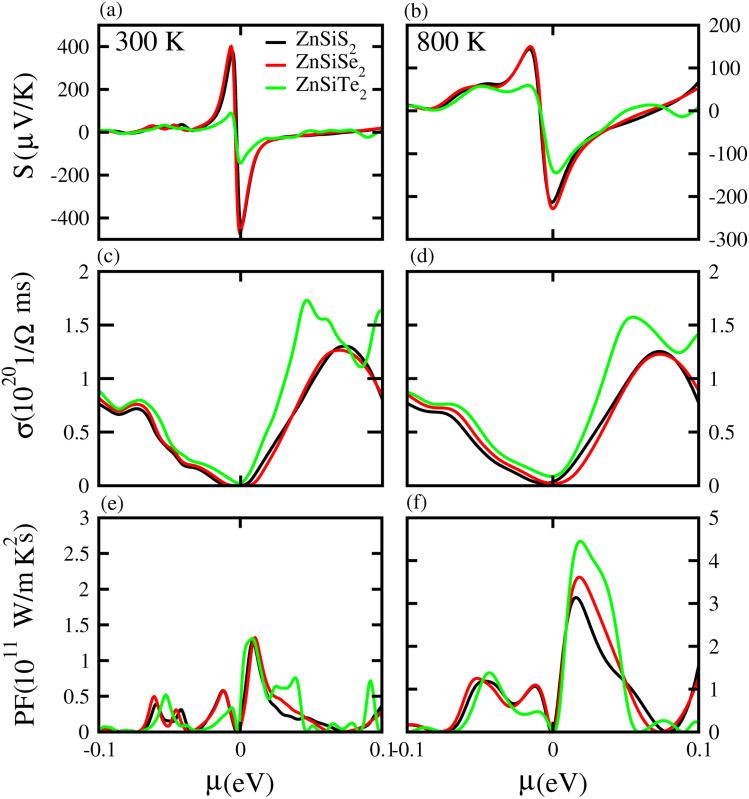
Seebeck coefficient (a, b), electrical conductivity (c, d), and power factor (e, f) results for ZnSiS_2_, ZnSiSe_2_ and ZnSiTe_2_ monolayers at 300 K and 800 K, respectively.

**Fig. 8:**
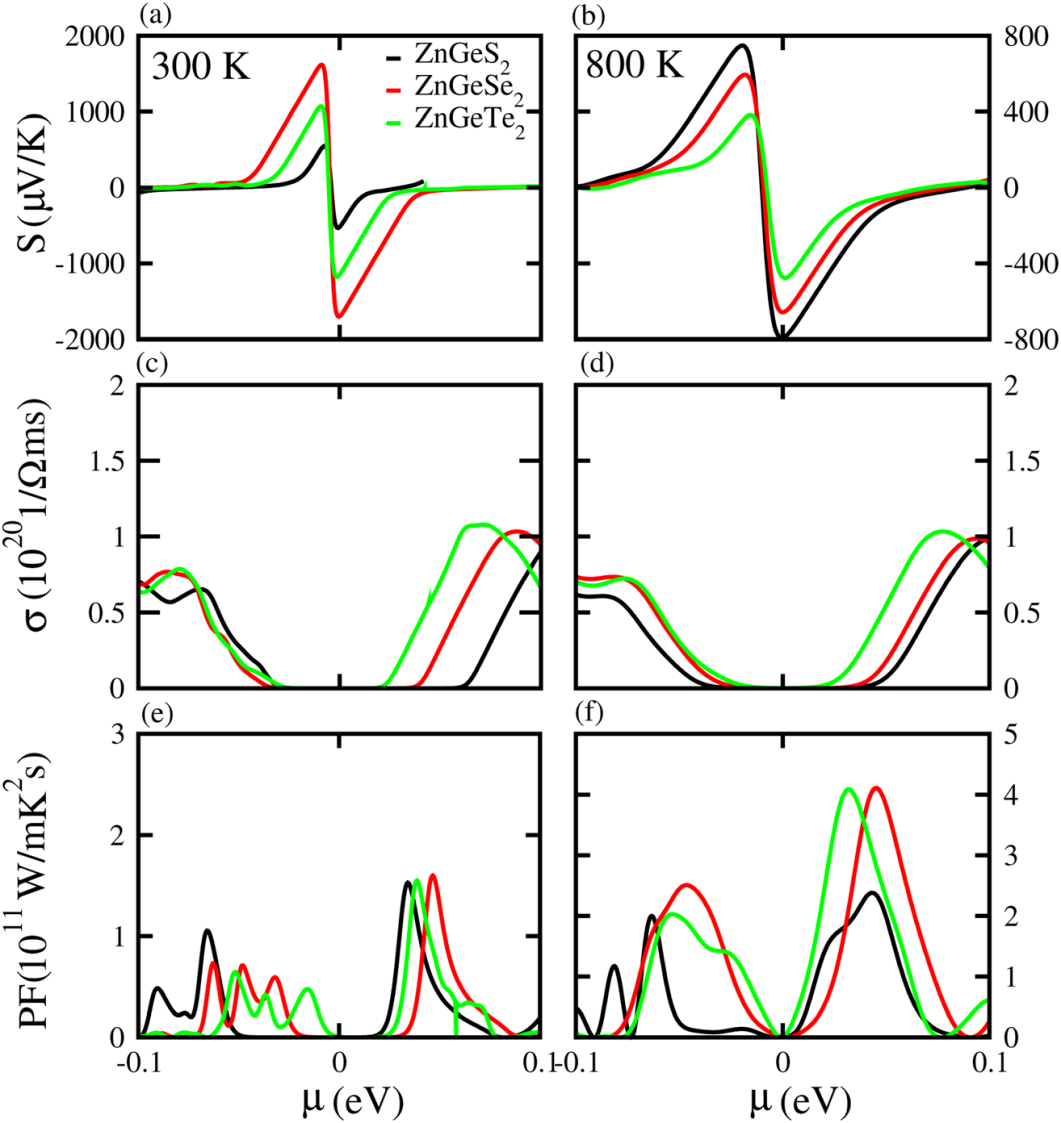
Seebeck coefficient (a, b), electrical conductivity (c, d), and power factor (e, f) results for ZnGeS_2_, ZnGeSe_2_ and ZnGeTe_2_ monolayers at 300 K and 800 K, respectively.

**Fig. 9:**
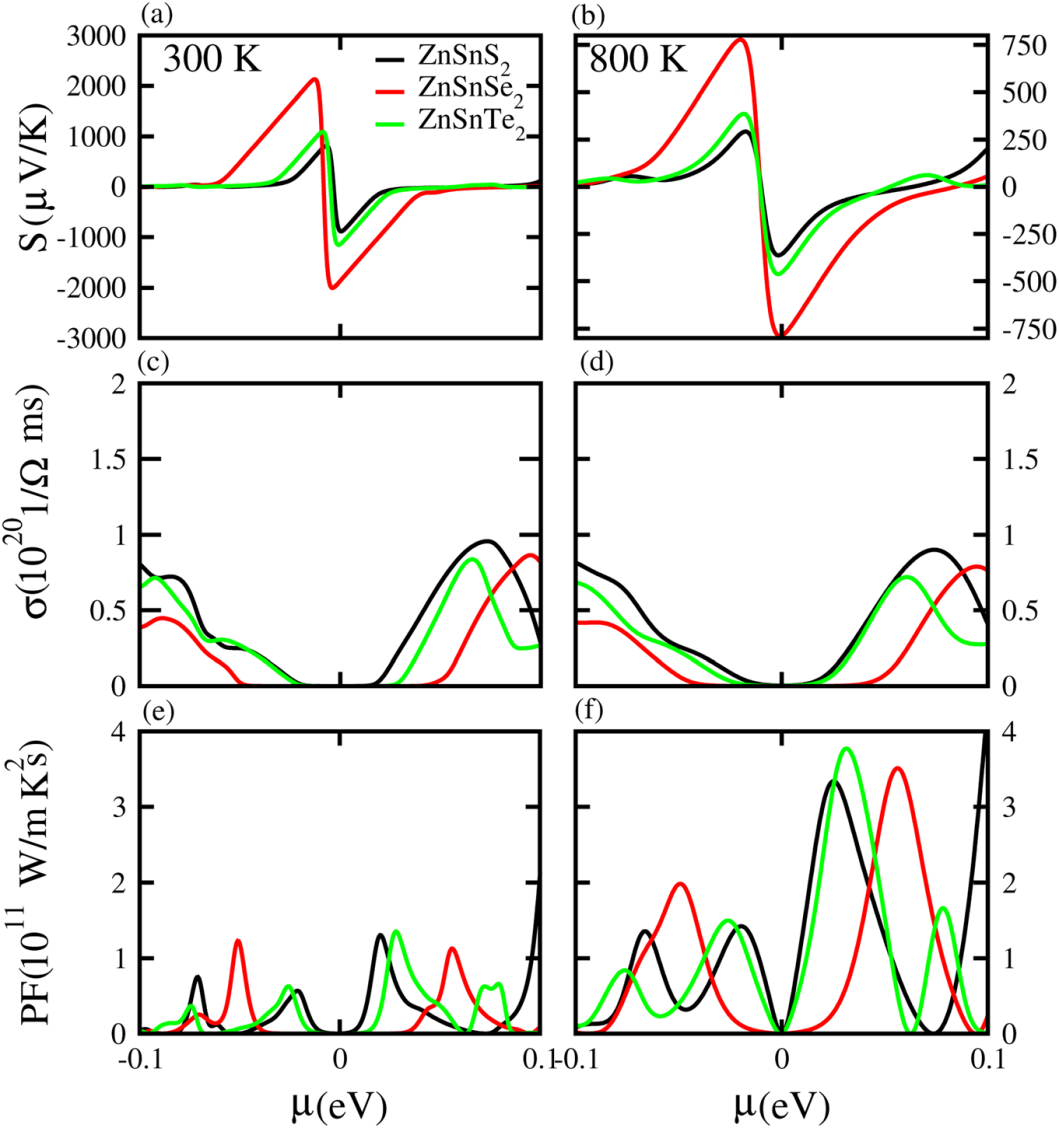
Seebeck coefficient (a, b), electrical conductivity (c, d), and power factor (e, f) results for ZnSnS_2_, ZnSnSe_2_ and ZnSnTe_2_ monolayers at 300 K and 800 K, respectively.

Based on the Boltzmann theory,^41^ we calculated transport coefficients such as the Seebeck coefficient, electrical conductivity, and power factor against chemical potential. The Seebeck coefficient is a quantified measurement of convinced voltage,^42^ and the Seebeck coefficient of a material represents the magnitude of the thermoelectric voltage induced by a temperature difference across the material.^45^ The Seebeck coefficient at (a) 300 K and (b) 800 K for ZnSiS_2_ (black), ZnSiSe_2_ (red) and ZnSiTe_2_ (green) is presented in Fig. 7 plotted against the chemical potential, and the Seebeck coefficient values for the n-type doped region are given in Table 2 and those for the p-type doped region are given in Table 3. The Seebeck coefficient results for ZnGeS_2_, ZnGeSe_2_ and ZnGeTe_2_ are shown in Fig. 8, and the values for the n-type doped region are given in Table 2 and those for the p-type doped region are given in Table 3. The Seebeck coefficient results for ZnSnS_2_, ZnSnSe_2_ and ZnSnTe_2_ are shown in Fig. 9, and the values for the n-type doped region are given in Table 2 and those for the p-type doped region are given in Table 3. From our calculations, it is clear that ZnSnSe_2_ has a greater value in both the n-type and p-type regions at 300 K and 800 K. According to the preceding discussion, ZnXY_2_ monolayers are p-type materials, and raising the temperature reduces the Seebeck coefficient, which makes these materials ideal for thermoelectric device applications, and similar results are also reported in ref. 43 and 44. The electrical conductivity (*σ*) of the materials is due to the holes and electrons in the semiconductors, and values of the electrical conductivity were calculated and plotted against *μ* for the ZnMN_2_ (M = Si, Sn and Ge and N = S, Se and Te) monolayers. For good thermoelectric materials, we need high *σ* values. The calculated values for *σ* for both n-type and p-type doped regions are given in Tables 2 and 3, respectively, for 300 K and 800 K. Our results show that ZnSiTe_2_ had a larger value in both the n-type and p-type regions at 300 K and 800 K. According to the preceding discussions, ZnMN_2_ monolayers are p-type materials, and raising the temperature reduces the Seebeck coefficient. To measure the thermoelectric power of a material, the power factor (PF) is one of the best parameters to describe the efficiency of that material with the general formula PF = *σS*^2^, where *S* represents the Seebeck coefficient and *σ* is the electrical conductivity of the material. The calculated values of the power factor at 300 K and 800 K are shown in Fig. 7 for monolayers of ZnSiS_2_, ZnSiSe_2_, and ZnSiTe_2_, Fig. 8 for monolayers of ZnGeS_2_, ZnGeSe_2_, and ZnGeTe_2_, and Fig. 9 for monolayers of ZnSnS_2_, ZnSnSe_2_ and ZnSnTe_2_. The values are given in Tables 2 and 3. It is obvious from Fig. 7(e, f), 8(e, f) and 9(e, f) that the peak values of the PF at both 300 K and 800 K in the p-type region are greater than in the n-type region. Furthermore, it can be seen that ZnSnS_2_ has a large PF at 300 K and 800 K because of its smaller Seebeck coefficient and higher electrical conductivity than ZnSiTe_2_, making it a promising candidate for thermoelectric applications.^48^

**Table 3:** Calculated values of the Seebeck coefficient (*S*), electrical conductivity (*σ*) and power factor (PF) at 300 K and 800 K for the n-type doped region of ZnSiS_2_, ZnSiSe_2_, ZnSiTe_2_, ZnGeS_2_, ZnGeSe_2_, ZnGeTe_2_, ZnSnS_2_, ZnSnSe_2_ and ZnSnTe_2_ monolayers.

Monolayers	300 K			800 K		
	*S* (μV/K)	*σ* (1/Ω ms)	PF (Wm K^2^ s)	*S* (μV/K)	*σ* (1/Ω ms)	PF (Wm K^2^ s)
ZnSiS_2_	380	1.25 × 10^20^	1.25 × 10^11^	140	1.26 × 10^20^	3.02 × 10^11^
ZnSiSe_2_	400	1.30 × 10^20^	1.32 × 10^11^	150	1.24 × 10^20^	3.80 × 10^11^
ZnSiTe_2_	100	1.75 × 10^20^	1.34 × 10^11^	50	1.57 × 10^20^	4.4 × 10^11^
ZnGeS_2_	500	0.9 × 10^20^	1.50 × 10^11^	700	1.00 × 10^20^	2.2 × 10^11^
ZnGeSe_2_	1500	1.00 × 10^20^	1.60 × 10^11^	500	1.09 × 10^20^	4.00 × 10^11^
ZnGeTe_2_	1000	1.10 × 10^20^	1.50 × 10^11^	1000	1.09 × 10^20^	4.00 × 10^11^
ZnSnS_2_	500	1.00 × 10^20^	1.35 × 10^11^	200	0.90 × 10^20^	2.20 × 10^11^
ZnSnSe_2_	2200	1.80 × 10^20^	1.10 × 10^11^	800	0.80 × 10^20^	2.00 × 10^11^
ZnSnTe_2_	1000	0.09 × 10^20^	1.45 × 10^11^	350	0.75 × 10^20^	2.50 × 10^11^

## IV. Conclusions

Using DFT, the structural, electronic, optical and thermoelectric properties of novel 2D Janus ZnMN_2_ (M = Si, Ge, Sn and N = S, Se, and Te) monolayers constructed according to their isoelectronic mutation have been investigated. Moreover, a direct band gap was observed in ZnSiS_2_, ZnSiSe_2_ and ZnSiTe_2_ monolayers, while ZnGe_2_, ZnGeSe_2_, ZnGeTe_2_, ZnSnS_2_, ZnSnSe_2_ and ZnSnTe_2_ possessed an indirect band gap. The electronic band structures revealed the semiconducting nature of the monolayers. Furthermore, values for the imaginary part of the dielectric function were calculated, which indicated strong modifications in the positions of excitons from the S atom to the Te atom, suggesting that this is promising for controlling the exciton–phonon interactions. Finally, we investigated the thermoelectric properties, including the Seebeck coefficient (*S*), electrical conductivity (*σ*), and power factor (PF), at temperatures of 300 K and 800 K, based on Boltzmann theory. The results confirmed that the peak values of the PF at 300 K and 800 K in the n-type region are greater than in the p-type region. Furthermore, it can be seen that ZnSiTe_2_ has a large PF at 300 K and 800 K because of its smaller Seebeck coefficient and higher electrical conductivity than ZnSnS_2_, making it a promising candidate for thermoelectric applications.

## Conflicts of interest

There are no conflicts to declare.

## Supplementary Material

## References

[cit1] Zhang G., Zhang Y. W. (2015). Mech. Mater..

[cit2] Chen Z. G., Han G., Yang L., Cheng L., Zou J. (2012). Prog. Nat. Sci.: Mater. Int..

[cit3] Zhu T., Liu Y., Fu C., Heremans J. P., Snyder J. G., Zhao X. (2017). Adv. Mater..

[cit4] Snyder G. J., Toberer E. S. (2008). Nat. Mater..

[cit5] Rull-Bravo M., Moure A., Fernandez J. F., Martin-Gonzalez M. (2015). RSC Adv..

[cit6] Sundarraj P., Maity D., Roy S. S., Taylor R. A. (2014). RSC Adv..

[cit7] Tan G., Zhao L.-D., Kanatzidis M. G. (2016). Chem. Rev..

[cit8] Gayner C., Kar K. K. (2016). Prog. Mater. Sci..

[cit9] Zhang G., Zhang Y.-W. (2017). J. Mater. Chem. C.

[cit10] Li D., Luo C., Chen Y., Feng D., Gong Y., Pan C., He J. (2019). ACS Appl. Energy Mater..

[cit11] Ge Z.-H., Zhao L.-D., Wu D., Liu X., Zhang B.-P., Li J.-F., He J. (2016). Mater. Today.

[cit12] Ortega S., Ibanez M., Liu Y., Zhang Y., Kovalenko M. V., Cadavid D., Cabot A. (2017). Chem. Soc. Rev..

[cit13] Kroon R., Mengistie D. A., Kiefer D., Hynynen J., Ryan J. D., Yu L., Muller C. (2016). Chem. Soc. Rev..

[cit14] Betal A., Bera J., Sahu S. (2021). Comput. Mater. Sci..

[cit15] Mishra P., Singh D., Sonvane Y., Ahuja R. (2020). Sustainable Energy Fuels.

[cit16] Novoselov K. S., Geim A. K., Morozov S. V., Jiang D., Zhang Y., Dubonos S. V., Grigorieva I. V., Firsov A. A. (2004). Sci. Adv..

[cit17] Yuan S., Zhou Q., Li S. (2018). Nano Lett..

[cit18] Wei Z., Zhu W., Wang F. (2018). Small Methods.

[cit19] Zhang T. T., Liang Y., Guo H., Fan H., Tian X. (2022). Appl. Surf. Sci..

[cit20] Demirci S. S., Avazlı N., Durgun E., Cahangirov S. (2017). Phys. Rev. B.

[cit21] Hu Z., Ding Y., Hu X., Zhou W., Yu X., Zhang S. (2019). Nanotechnology.

[cit22] Naguib M., Mashtalir O., Lukatskaya M. R., Dyatkin B., Zhang C., Presser V., Gogotsi Y., Barsoum M. W. (2014). Chem. Commun..

[cit23] Lu A.-Y., Zhu H., Xiao J., Chuu C.-P., Han Y., Chiu M.-H., Cheng C.-C., Yang C.-W., Wei K.-H., Yang Y. (2017). Nat. Nanotechnol..

[cit24] Zhang T., Liang Y., Guo H., Fan H., Tian X. (2022). Appl. Surf. Sci..

[cit25] Zhao L.-D., Lo S.-H., Zhang Y., Sun H., Tan G., Uher C., Wolverton C., Dravid V. P., Kanatzidis M. G. (2014). Nature.

[cit26] Chang C., Wu M., He D., Pei Y., Wu C.-F. (2018). et al.. Science.

[cit27] Babaei H., Khodadadi J. M., Sinha S. (2014). Appl. Phys. Lett..

[cit28] Guo S.-D., Li Y.-F., Guo X.-S. (2019). Comput. Mater. Sci..

[cit29] Bera J., Betal A., Sahu S. (2021). J. Alloys Compd..

[cit30] Giannozzi P., Baroni S., Bonini N., Calandra M., Car R., Cavazzoni C., Ceresoli D., Chiarotti G. L., Cococcioni M., Dabo I., Dal Corso A., de Gironcoli S., Fabris S., Fratesi G., Gebauer R., Gerstmann U., Gougoussis C., Kokalj A., Lazzeri M., Martin-Samos L., Marzari N., Mauri F., Mazzarello R., Paolini S., Pasquarello A., Paulatto L., Sbraccia C., Scandolo S., Sclauzero G., Seitsonen A. P., Smogunov A., Umari P., Wentzcovitch R. M. (2009). J. Phys.: Condens. Matter.

[cit31] Perdew J. P., Burke K., Ernzerhof M. (1996). Phys. Rev. Lett..

[cit32] Perdew J. P., Burke K., Ernzerhof M. (1998). Phys. Rev. Lett..

[cit33] Yousuf S., Gupta D. C. (2019). Results Phys..

[cit34] Ahmad I., Khan S. A., Idrees M., Haneef M., Shahid I., Din H. U., Khan S. A., Amin B. (2018). Phys. B.

[cit35] Amin B., Kaloni T. P., Schwingenschlögl U. (2014). RSC Adv..

[cit36] Alam Q., Muhammad S., Idrees M., Hieu N. V., Binh N. T. T., Nguyen C., Amin B. (2021). RSC Adv..

[cit37] Perdew J. P., Burke K., Ernzerhof M. (1996). Phys. Rev. Lett..

[cit38] Heyd J., Scuseria G. E., Ernzerhof M. (2003). J. Chem. Phys..

[cit39] Din H. U., Idrees M., Alam Q., Amin B. (2021). Appl. Surf. Sci..

[cit40] He X., Singh D. J., Boon-On P., Lee M.-W., Zhang L. (2018). J. Am. Chem. Soc..

[cit41] He X., Luo L.-S. (1997). Phys. Rev. E: Stat., Nonlinear, Soft Matter Phys..

[cit42] Zhu Q., Kim H. S., Ren Z. (2017). Rev. Sci. Instrum..

[cit43] Khan F., Din H. U., Khan S. A., Rehman G., Bilal M., Nguyen C. V., Ahmad I., Gan L.-Y., Amin B. (2019). J. Phys. Chem. Solids.

[cit44] Bashir K., Bilal M., Amin B., Chen Y., Idrees M. (2023). RSC Adv..

[cit45] Zhou Z., Uher C. (2005). Rev. Sci. Instrum..

[cit46] Olmon R. L., Slovick B., Johnson T. W., Shelton D., Oh S.-H., Boreman G. D., Raschke M. B. (2012). Phys. Rev. B: Condens. Matter Mater. Phys..

[cit47] Müller M., Budde W., Gottfried-Gottfried R., Hübel A., Jähne R., Kück H. (1996). Sens. Actuators, A.

[cit48] Lv H. Y., Lu W. J., Shao D. F., Lu H. Y., Sun Y. P. (2016). J. Mater. Chem. C.

